# Rhabdomyolysis caused by *Botrychium ternatum* intoxication: Case report and literature review

**DOI:** 10.1097/MD.0000000000037304

**Published:** 2024-03-01

**Authors:** Ming-Wei Liu, Chun-Hai Zhang, Qiu-Juan Zhang, Bing-Ran Zhang

**Affiliations:** a Department of Emergency, People's Hospital of Dali Bai Autonomous Prefecture, Dali, Yunnan, China; b Department of Emergency, The First Affiliated Hospital of Kunming Medical University, Kunming, Yunnan, China.

**Keywords:** *Botrychium ternatum*, case report, diagnosis, rhabdomyolysis, therapeutical intervention

## Abstract

**Rationale:**

*Botrychium ternatum* ((Thunb.) Sw.), a traditional Chinese medicine, is known for its therapeutic properties in clearing heat, detoxifying, cough suppression, and phlegm elimination. It has been extensively used in clinics for the treatment of many inflammation-related diseases. Currently, there are no documented cases of rhabdomyolysis resulting from *Botrychium ternatum* intoxication.

**Patient concerns:**

A 57-year-old male presented with a complaint of low back discomfort accompanied by tea-colored urine lasting for 4 days. The patient also exhibited markedly increased creatine phosphate kinase and myoglobin levels. Prior to the onset of symptoms, the patient consumed 50 g of *Botrychium ternatum* to alleviate pharyngodynia.

**Diagnoses:**

The patient was diagnosed with rhabdomyolysis due to *Botrychium ternatum* intoxication.

**Interventions:**

The patient underwent a substantial volume of fluid resuscitation, diuresis, and alkalization of urine, as well as correction of the acid-base balance and electrolyte disruption.

**Outcomes:**

Following a 10-day treatment plan involving massive fluid resuscitation, diuresis, and alkalization of urine, the patient showed notable improvement in his lower back pain and reported the absence of any discomfort. Following reexamination, the levels of creatine phosphate kinase and myoglobin were restored to within the normal ranges. Additionally, no abnormalities were detected in liver or renal function. As a result, the patient was considered eligible for discharge and was monitored.

**Conclusions:**

*Botrychium ternatum* intoxication was associated with the development of rhabdomyolysis. To manage this condition, it is recommended that patients provide massive fluid resuscitation, diuresis, alkalization of urine, and other appropriate therapeutic interventions.

**Lesson:**

Currently, there are no known cases of rhabdomyolysis resulting from *Botrychium ternatum* intoxication. However, it is important to consider the potential occurrence of rhabdomyolysis resulting from *Botrychium ternatum* intoxication when there is a correlation between the administration of *Botrychium ternatum* and the presence of muscular discomfort in the waist or throughout the body, along with tea-colored urine. Considering the levels of creatine phosphate kinase and myoglobin, the diagnosis or exclusion of rhabdomyolysis caused by *Botrychium ternatum* intoxication should be made, and suitable treatment should be administered accordingly.

## 1. Introduction

Rhabdomyolysis (RM) is a pathological condition characterized by the rupture of skeletal muscle fibers and subsequent lysis of striated muscle cells for various etiological reasons. This process leads to the release of a significant quantity of intracellular muscle components into the systemic circulation, which possesses toxic properties and can inflict damage to tissues and organs.^[1]^ The etiology of RM is multifaceted and can be broadly categorized into mechanical and nonmechanical determinants. Mechanical factors that contribute to muscle disorders include trauma, constriction, excessive exercise, and continuous muscle contraction. Conversely, non-mechanical factors include water and electrolyte disorders, endocrine diseases, excessive use of drugs and poisons, viral or bacterial infections, polymyositis, and metabolic myopathy resulting from genetic defects.^[2]^ Trauma and drug usage are recognized as the prevalent etiological factors contributing to the occurrence of RM in adults. Clinically, drugs commonly cause RM, including HMG-CoA reductase inhibitors such as simvastatin and prvastatin, as well as the β2 receptor agonist terbutaline.^[3,4]^ In children, infection is a common cause contributing to the occurrence of RM, constituting approximately one-third of all cases.^[5]^ The clinical manifestation of RM is atypical. Muscle pain, weakness, and edema were the most frequently observed symptoms. However, it is noteworthy that up to 50% of patients do not experience concurrent muscle pain and weakness,^[1]^ resulting in a missed diagnosis when the symptoms are minor.

*Botrychium ternatum* (Thunb.) Sw. is a traditional medicinal plant of the genus *Botrychiaceae* and the genus *Botrychium Sw* and is known as Xiaochunhua, Yiduoyun, Huajue, and Dulijinji. It has been traditionally used for heat-clearing and detoxification, suppressing hyperactive liver to alleviate endogenous wind-related symptoms such as dizziness and headache, cough-relieving, hemostasis, and vision-enhancing.^[6]^ In China, it has been used for the clinical management of inflammatory disorders. Currently, there is no documented evidence of RM resulting from *Botrychium ternatum* intoxication.

## 2. Case report

### 2.1. Ethics approval and consent to participate

Informed written consent was obtained from the patient for publication of this case report and accompanying images.

This study was reviewed and approved by the local ethics committee of the First Affiliated Hospital of Kunming Medical University. The procedures were performed in accordance with the Helsinki Declaration of 1975, revised in 2000.

### 2.2. Medical history

A 57-year-old male experienced pharyngodynia following the ingestion of 30 g of *Botrychium ternatum* 4 days ago. The patient sought medical treatment at the First Affiliated Hospital of Kunming Medical University due to a 4-day history of low back pain accompanied by tea-colored urine. The patient reported the absence of fever, chest pain, chest tightness, urgent urination, frequent urine, strenuous exercise, wheezing, or dyspnea.

### 2.3. Past medical history

The patient underwent kidney transplantation at the First Affiliated Hospital of Kunming Medical University in October 2012. The patient consistently adhered to a regimen of anti-transplantation medications and underwent annual renal function assessments, which yielded normal results. The patient had no prior medical conditions related to hypertension, diabetes, cardiovascular, cerebrovascular, pulmonary, nutritive, endocrine, or other significant organ disorders or infectious diseases. The patient exhibited a lack of reported trauma, surgical procedures, blood transfusions, and allergies, as well as information regarding vaccination history.

### 2.4. Physical examination

The patient body temperature was 36.4°C, pulse rate was 82 beats per minute, respiratory rate was 19 breaths per minute, and blood pressure was 143/92 mm Hg. The overall physical state appeared to be normal, since there was no yellowing in the skin sclera and no enlargement of the superficial lymph nodes throughout the body. There were no facial abnormalities, and both eyeballs exhibited typical characteristics. Hypertrophy of the thyroid gland was not observed. No anomalies were detected in the chest region during chest auscultation. Respiratory sounds were thick in both lungs, without the presence of dry or wet rales. Furthermore, there was no evidence of cardiac border enlargement, with a heart rate of 87 beats/min. In addition, no pathological murmurs were detected in any of the valve areas. The abdominal region exhibits a flat and soft appearance and is devoid of tenderness or rebound pain. The liver remained unaffected, whereas bowel sounds were within the expected range. Muscle strength and tension in the limbs were within the normal parameters, and no pathological indications were elicited.

### 2.5. Laboratory data

Laboratory tests were conducted on September 4, 2023. The results of the analysis revealed the following values: white blood cell count (WBC), 9.79 × 10^9^/L; neutrophils (N), 78.3%; lymphocytes (L), 9.9%; red blood cell count (RBC), 6.43 × 10^12^/L; hemoglobin (Hb), 190 g/L; platelet count (PLT), 349 × 10^9^/L; plateletcrit (PCT), < 0.05 mg/L; and C-reactive protein (CRP), 26.5pg/L. The levels of myocardial enzymes observed in the patients’ blood samples were as follows: creatine phosphate kinase (CK), 7683.9 U/L; creatine phosphate kinase-MB (CK-MB), 173.40 U/L; lactate dehydrogenase (LDH), 1132 U/L; α-hydroxybutyrate dehydrogenase (α-HBDH), 883 U/L; myoglobin, 2144.53 ng/mL; and troponin, 0.016 ng/mL. The results of the liver function tests reveal that alanine aminotransferase, 26.28U/L; aspartate aminotransferase (AST), 62.33U/L; total bilirubin (TBIL), 10.9μmol/L; direct bilirubin (DBIL), 3.5μmol/L; and indirect bilirubin (DBIL), 7.4μmol/L. Renal function parameters were as follows: urea nitrogen, 6.61μmol/L; creatinine, 105.31μmol/L. The electrolyte composition includes Na^+^, 142.34 mmol/L; K^+^, 3.59 mmol/L; Cl^-^, 111.75 mmol/L; Ca^++^, 2.28 mmol/L. The results of the coagulation and fibrinolytic system tests showed that routine urine tests, tuberculosis antibodies, parasite antibodies, systemic lupus erythematosus, rheumatoid-associated antibodies, anti-cardiolipin antibodies, and anti-neutrophil cytoplasmic antibodies yielded negative results. The nucleic acid test for the novel coronavirus was negative.

### 2.6. Medical imagology

**Figure 1.**
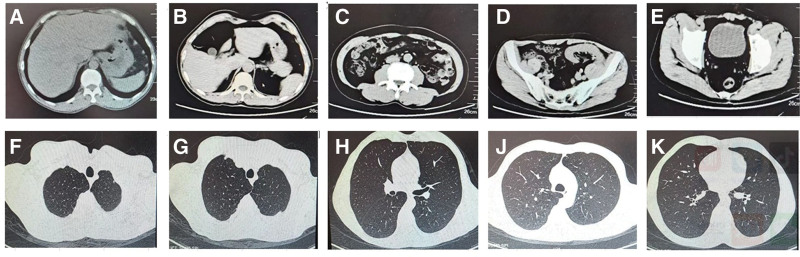
Changes of chest and abdomen CT at admission. (A–E) Changes of Abdominal CT at admission. (F–I) Changes of chest CT at admission.

On September 18, 2023, chest computed tomography (CT) revealed numerous pulmonary nodules in both lungs, measuring approximately 2 to 8 mm in diameter. The CT scan also identified the presence of emphysema, as well as several calcifications in the aortic and coronary artery walls (Fig. 1F–K), while no evident infection lesions were observed (Fig. 1F–K). Abdominal CT tomography revealed no discernible anomalies in the liver, gallbladder, spleen, or pancreas (Fig. 1A–E). The kidney that underwent transplantation was situated within the right iliac fossa and contained numerous tiny stones (Fig. 1A–E). No apparent thickening of the appendix was observed (Fig. 1A–E).

### 2.7. Diagnosis and treatment

Based on the patient medical history, presenting symptoms and signs, as well as reported usage of *Botrychium ternatum*, concomitant shoulder and back muscle pain, notable elevations in serum creatine phosphate kinase and myoglobin levels, ruling out any influence from external strenuous exercise, parasitic infection, or rheumatic immune-related conditions, the patient was diagnosed with RM due to *Botrychium ternatum* intoxication. The patient was treated with large-volume fluid resuscitation (approximately 10 L per day), along with diuresis. Additionally, therapeutic interventions were performed to restore acid-base balance and electrolyte disturbances. The patient condition was closely monitored through dynamic observation of creatine phosphate kinase and myoglobin levels as well as liver and kidney function and electrolyte fluctuations. After 9 days of treatment, there was a notable reduction in the levels of creatine phosphate kinase and myoglobin, approaching values considered within the normal range (Table 1). Additionally, the liver and renal functions were normal (Table 1). The patient was discharged after further monitoring and examination.

**Table 1:** Changes of creatine phosphate kinase, myoglobin, and liver and kidney function before and after treatment.

	At admission	3 D after treatment	6 D after treatment	9 D after treatment	2 W after discharge
CK (1U/L)	7683.9	3167.14	568.35	328.49	257.18
MYO (ng/mL)	2144.53	2209.62	997.36	107.54	61.48
ALT(U/L)	26.28	24.97	29.74	26.57	27.31
AST(U/L)	62.33	57.04	68.13	60.71	56.03
BUN (μmol/L)	6.61	5.93	5.17	6.39	5.82
Cr (μmol/L)	105.7	111.8	109.4	104.8	106.5
Na^+^ (mmol/L)	142.34	139.52	146.3	142.4	146.2
K^+^(mmol/L)	3.59	3.48	4.67	4.49	4.17
Cl^-^(mmol/L)	111.75	109.41	106.8	102.63	108.74
Ca^++^(mmol/L)	2.28	2.14	2.09	2.37	2.53

ALT = alanine aminotransferase, AST = aspartate aminotransferase, BUN = urea nitrogen, CK = creatine phosphate kinase, Cr = creatinine, MYO = myoglobin.

### 2.8. Follow-up after treatment

The patient exhibited no signs of discomfort during the 2-week follow-up period after discharge. Furthermore, reexamination revealed no abnormalities in creatine phosphate kinase, myoglobin, or liver and kidney function (Table 1). The patient was informed that administration of *Botrychium ternatum* would be contraindicated in the future.

## 3. Discussion

*Botrychium ternatum*, also referred to as Yiduoyun, Beishesheng, Sanxuecao, Potianyun, Xiaochunhua, Shebujian, Liangqixixin, Dujiaojinji, Dujiaohao, Dongcao, Huangliangqi, and Jizhualian, is a traditional medicinal plant and a Chinese herbal medicine belonging to the *Botrychiaceae* genus and the *Botrychium* Sw genus.^[7]^ *Botrychium ternatum* is typically found in shaded areas of shrubland ecosystems, particularly at elevations ranging from 400 to 1000 m above sea level. It is distributed in several regions of China, including Zhejiang, Jiangsu, Anhui, Jiangxi (specifically, Lushan), Fujian, Hunan, Hubei, Guizhou, Sichuan, and Taiwan. It is also found in Japan, Korea, Vietnam, and the Himalayas.^[8]^ It contains various chemical components, such as flavonoids, phenolic acids, terpenoids, alkaloids, polysaccharides, and lactones others.^[9]^ Multiple studies have provided evidence that it has good anti-inflammatory, antibacterial, antiviral, anti-allergic, and anti-oxidative effects and can modulate the immune system of the body.^[10–12]^ Additionally, ternatin has demonstrated efficacy in counteracting cell proliferation induced by chemogenic carcinogens.^[10]^ Clinically, it exhibits potential for treating several ailments, including pharyngodynia, external fever, urinary tract infections, and snake and insect bites. Additionally, it possesses health-enhancing properties, such as promoting mental tranquility, strengthening the spleen, and exerting a relaxing effect on the liver while reducing blood pressure. The patient had a history of using 50 g of *Botrychium ternatum* the day before the onset of the disease.

Currently, there are no unified diagnostic criteria for RM. The primary clinical diagnostic criterion for RM is elevated creatine phosphate kinase levels. However, it is important to note that different studies have employed different diagnostic thresholds for this criterion.^[1]^ Chavez and Stahl conducted a comprehensive analysis of the existing literature on the definition of RM published between 2006 to 2015 and 1968 to 2018, respectively. They revealed that a majority of the studies considered a creatine phosphate kinase level >200 IU/L as the critical value, and commonly selected a creatine phosphate kinase level exceeding 5 times the critical value, 1000IU/L, as the diagnostic criteria for RM.^[13,14]^ Myoglobin serves as a crucial biomarker for the early detection and prognosis of RM.^[15]^ Following the administration of *Botrychium ternatum* at a dose of 50 g over a period of 4 days, the patient presented with symptoms of low back pain accompanied by the presence of tea-colored urine. Examination of the patient venous blood revealed that the levels of creatine phosphate kinase and myoglobin had significantly increased to 7184 U/L and 2231 pg/L, respectively. The patient did not exhibit any indications of hypertension, diabetes, renal injury, or a related family genetic history of these conditions. The patient diagnosis of RM attributed to *Botrychium ternatum* was determined based on the absence of a history of strenuous exercise, severe infection, fever, and medication usage prior to the commencement of the condition. In addition, AST and ALP levels in patients were significantly elevated; therefore, acute liver injury induced by RM was considered. Research has indicated that a notable proportion of patients diagnosed with RM (25%) have signs of liver impairment.^[16]^ Myoglobin decomposition leads to the generation of a significant quantity of heme, causing the heme-binding protein to become saturated and leading to the buildup of heme in the plasma. Heme has the capacity to function as both a pro-oxidant and pro-inflammatory substance,^[17]^ which can lead to acute kidney injury (AKI) and liver injury. The incidence of AKI in patients with RM ranges from 7% to 10%, and RM accounts for 5% to 15% of all instances of AKI.^[16]^ There is a prevailing consensus that there is a positive correlation between the level of creatine phosphate kinase and the risk of AKI. When the level of creatine phosphate kinase ranges from 5000 U/L to 15,000 U/L, the likelihood of AKI is approximately 35%. However, when creatine phosphate kinase levels exceed 15,000 U/L, the risk of AKI increases to > 70%.^[16]^ Hence, although the patient did not currently exhibit AKI, there remains potential for its development during the course of treatment.

The key to the treatment of RM is to identify the cause of RM and promptly deal with the associated risk factors. Common therapeutic interventions consist of administering intravenous fluids, correcting electrolyte disorders, and kidney replacement therapy. When RM is suspected, the primary objective is to prevent the occurrence of AKI. The occurrence of prerenal azotemia is unavoidable because of intermuscular edema and reduced blood volume during RM.^[18]^ The initial treatment of azotemia is rapid fluid resuscitation at a rate of 1.5L/h^[18]^ with the treatment goals of achieving a urine volume of 200mL/h, urine pH > 6.5, and plasma pH < 7.5.^[18]^ The use of balanced salt or isotonic saline is recommended for the selection of intravenous fluids. It is imperative to conduct a thorough assessment of the volume status and closely monitor urine volume to prevent complications, such as excessive fluid retention and metabolic acidosis.^[19]^ Owing to the potential development of hyperchloremic acidosis, it is reasonable to administer both saline and sodium bicarbonate to patients with RM, particularly those exhibiting acidosis. During treatment, it is imperative to closely evaluate many physiological parameters including urine pH, serum sodium bicarbonate concentration, blood calcium levels, and blood potassium levels. In cases where the urine pH value fails to increase during sodium bicarbonate treatment for 4 to 6 hours, or if symptoms indicative of hypocalcemia manifest, it is advised to discontinue the administration of sodium bicarbonate and instead proceed with standard saline treatment.^[20]^ The prevention of RM-associated AKI was the subject of a meta-analysis consisting of 27 trials, which yielded several recommendations^[21]^: prompt administration of intravenous fluids, ideally within 6 hours following muscle injury; urine volume of 300 mL/h or more maintained for at least the first 24 hours; intravenous administration of sodium bicarbonate only limited to correcting systemic acidosis; after massive fluid resuscitation, mannitol should only be utilized to sustain a urine volume of 300 mL/h or higher; and there is insufficient evidence to support the preference for mannitol over sodium bicarbonate. Therefore, the patient underwent treatment involving administration of a substantial volume of fluid resuscitation, approximately 10 L per day, along with adequate diuretic therapy, correction of acid-base imbalances, and management of electrolyte disturbances.

## 4. Strengths and limitations of the study

Strengths: Intoxication caused by *Botrychium ternatum* leads to RM, a condition that can be effectively managed with fluid resuscitation, diuretic therapy, alkalization of urine, and other appropriate treatment interventions.

Limitations: The precise etiology of RM resulting from *Botrychium ternatum* intoxication remains unclear and requires further investigation. The occurrence of RM as a result of *Botrychium ternatum* intoxication is relatively small, and additional verification through multicenter clinical trials with large sample sizes is still required. The necessity of mannitol administration in intravenous fluid replacement and the process of selecting appropriate intravenous fluids are the subjects of ongoing debate, necessitating further validation studies.

## 5. Conclusion

This case report suggests that ingestion of *Botrychium ternatum* may result in the development of RM. In clinical practice, it is important to consider the possibility of RM caused by *Botrychium ternatum* intoxication when patients present with symptoms such as low back or generalized muscle pain accompanied by tea-colored urine following ingestion. To confirm or exclude the diagnosis of RM caused by *Botrychium ternatum* intoxication, prompt measurement of creatine phosphate kinase and myoglobin levels is recommended. Patients diagnosed with RM should be administered a substantial volume of fluid for rehydration purposes, along with diuretic treatment to promote urine production. Additionally, efforts should be made to alkalize the urine and safeguard the functioning of the liver and kidneys. In critically ill patients, the possibility of blood purification should be considered.

## Author contributions

**Conceptualization:** Ming-Wei Liu, Chun-Hai Zhang, Bing-Ran Zhang.

**Data curation:** Ming-Wei Liu, Qiu-Juan Zhang, Bing-Ran Zhang.

**Formal analysis:** Chun-Hai Zhang, Bing-Ran Zhang.

**Funding acquisition:** Ming-Wei Liu, Qiu-Juan Zhang.

**Investigation:** Ming-Wei Liu.

**Methodology:** Chun-Hai Zhang, Bing-Ran Zhang.

**Project administration:** Ming-Wei Liu, Qiu-Juan Zhang.

**Software:** Qiu-Juan Zhang.

**Supervision:** Ming-Wei Liu, Chun-Hai Zhang, Bing-Ran Zhang.

**Validation:** Qiu-Juan Zhang.

**Visualization:** Ming-Wei Liu.

**Writing – original draft:** Chun-Hai Zhang, Qiu-Juan Zhang.

**Writing – review & editing:** Ming-Wei Liu, Qiu-Juan Zhang, Bing-Ran Zhang.
